# Responsive fungal insoles for pressure detection

**DOI:** 10.1038/s41598-023-31594-9

**Published:** 2023-03-21

**Authors:** Anna Nikolaidou, Neil Phillips, Michail-Antisthenis Tsompanas, Andrew Adamatzky

**Affiliations:** 1 Unconventional Computing Laboratory, UWE, Bristol, UK; 2 Department of Architecture, UWE, Bristol, UK

**Keywords:** Biomaterials, Diagnosis, Quality of life

## Abstract

Mycelium bound composites are promising materials for a diverse range of applications including wearables and building elements. Their functionality surpasses some of the capabilities of traditionally passive materials, such as synthetic fibres, reconstituted cellulose fibres and natural fibres. Thereby, creating novel propositions including augmented functionality (sensory) and aesthetic (personal fashion). Biomaterials can offer multiple modal sensing capability such as mechanical loading (compressive and tensile) and moisture content. To assess the sensing potential of fungal insoles we undertook laboratory experiments on electrical response of bespoke insoles made from capillary matting colonised with oyster fungi *Pleurotus ostreatus* to compressive stress which mimics human loading when standing and walking. We have shown changes in electrical activity with compressive loading. The results advance the development of intelligent sensing insoles which are a building block towards more generic reactive fungal wearables. Using FitzHugh-Nagumo model we numerically illustrated how excitation wave-fronts behave in a mycelium network colonising an insole and shown that it may be possible to discern pressure points from the mycelium electrical activity.

## Introduction

In-shoe sensor technologies have been widely used in the clinical domain for disease detection, diagnostics and therapeutic use. Smart insoles can detect impairments in balance, gait, posture, muscle strength and cognition, providing valuable information about the user’s physical and mental health^1–3^. They most commonly integrate pressure or optical sensor technologies in relevant locations for monitoring the foot–ground interaction force^4^, providing complex functions and exhibiting broad sensing range when exposed to mechanical stimuli. As detailed in^4^ smart insoles can be divided into three subgroups: (1) passive smart insoles able to sense parameters including weight loading of user, local terrain topology, volatile organic compounds, (2) active or reactive smart insoles able to sense and react performing some actions, by integrating an actuator, (3) advanced smart insoles able to sense, react and tailor their behaviour to specific operating circumstances.

Integrating sensor technologies into insoles, patterns and strategies for executing different functional tasks can be assessed, capturing data that can form the basis for use in areas such as rehabilitation, prehabilitation, monitoring elderly people who have mobility problems, mitigating slipping and falling as well as assessing long-term chronic conditions such as Dementia, Parkinson’s disease and stroke^5^. For example, smart insoles have been used for the measurement of the weight pressure distribution that a patient exerts on each foot, in addition to the gait time, swing time, and stance time of each leg while walking to diagnose several medical conditions^6^.

Smart insoles have lately found new applications outside the clinical domain. The proliferation of consumer-grade smart wearables has further propelled the development of commercial in-shoe devices to assess health and wellness-related mobility parameters in activities of daily living^7–10^ e.g., pressure mapping of insoles in footwear based on conventional sensor technology (e.g. dielectric layer) have been reported^11,12^ and insoles with diagnostic capabilities are commercially available, for example NURVV Run Smart Insoles^13^. While the use of smart insoles in non-medical applications has recently attracted significant interest, there are important challenges to overcome.

Cost represents a limiting factor. The devices require a number of sensors and actuators driven by electrical circuit components, usually supplied by a battery, making them not only expensive to fabricate but also significantly contributing to the depletion of natural resources. Insoles based on conventional sensor technology have short battery life (e.g. 5 h^14^) and are available in a limited number of insole sizes/shapes (e.g. 6 sizes^15^). Moreover, the performance of smart insoles is directly linked to the number and distribution of the sensors integrated as well as the identification of optimal sensor locations that match areas subject to the highest plantar pressures during gait or standing^4^. These locations can vary depending on the foot sizes and gait patterns of different users and can therefore alter or limit the gait or standing event recognition accuracy.

Biomaterials, such as mycelium bound composites, present a promising alternative to conventional smart insoles. They exhibit sensing and responsive capabilities without requiring additional space (for support infrastructure) and external inputs (e.g., electrical power sources) to operate, using its own bioelectric activity. Fungal sensors offer increased biodegradability, they are self- sustainable as they can self-grow, self-repair and self-assemble, they are abundant and offer in situ low technology cultivation. Moreover, they present low capital requirements and are easily scalable for the production of customised insole sizes.

In our previous studies, we demonstrated that living blocks of colonised by filamentous polypore fungus *Ganoderma resinaceum* substrate (MOGU’s collection code 19–18, Mogu S.r.l., Inarzo, Italy), showed immediate responses in the form of spikes of electrical potential when subjected to weight application (8 kg and 16 kg), recognising the application or removal of weight^16^. In this paper, we present an illustrative scoping study in which we research the response of mycelium composite insoles to pressure generated by the feet during gait or standing. The primary objective of the studies reported in this paper is to assess the performance and spiking activity of the fungal insoles when exposed to mechanical stimuli and in particular, weight shifting (shifting of weight from toe to heel). We present a prototype of pressure-sensitive fungal insoles, aiming to open up opportunities for further research and discussions on the novel field of responsive smart insoles from living material, with the objective of enabling real-time applications and providing a sustainable, cost efficient and accurate tool for posture, gait and activity recognition events.

## Methods

**Figure 1:**
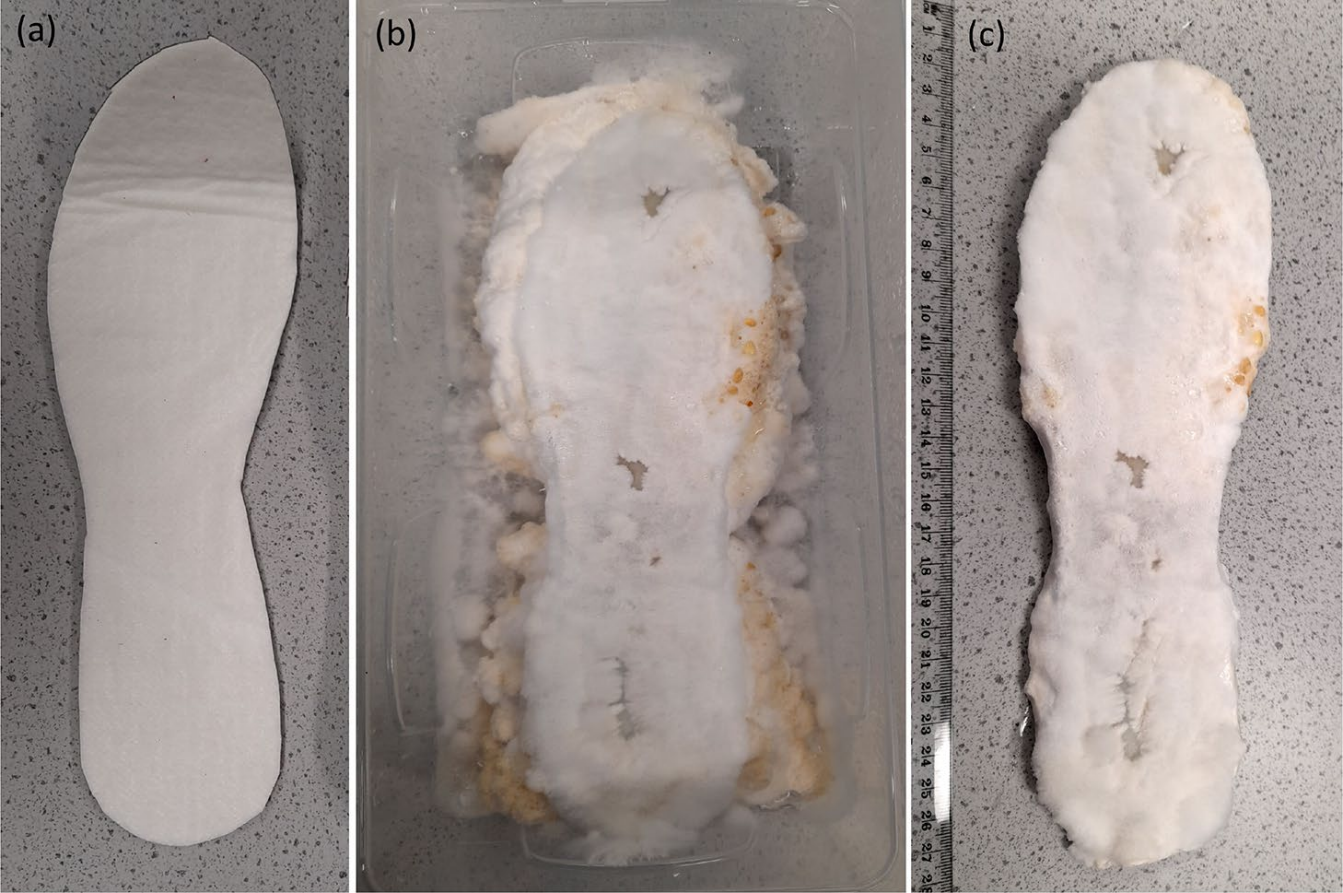
(**a**) capillary matting cut into insole pattern (**b**) insole on bed of spawn (**c**) well colonised insole.

An innovative new method of forming insoles colonised with fungi from capillary matting (rather than hemp) was developed through multiple iterations. In a sterilised hydroponic growing tent with a silver Mylar light proof inner lining (Green Box Tents, UK), 200 g slab of mixed (mostly Rye grain) seed substrate well colonised with *Pleurotus ostreatus* (Ann Miller’s Speciality Mushrooms, UK, https://www.annforfungi.co.uk/shop/oyster-grain-spawn/) was placed in the bottom of a sterilised clean plastic container (5 L, 280 $\times$ 145 $\times$ 110 mm, Amazon, UK). Here, it is noteworthy that *P. ostreatus* was selected based on its higher rate of substrate colonisation and trivial cultivation procedure. Multi-layered, absorbent, capillary action matting, $\sim$ 3 mm thickness, made from bonded, non-toxic, wool and acrylic fibres, $450\,\hbox {g}\,\hbox {m}^{-2}$ (manufactured by Tech-Garden, UK) was manually cut into the shape of insoles (UK ladies size 10) using hemp scissors (manufactured by Pemmiproducts, Germany), see Fig. 1a. The bespoke insole was sprayed with deionized water (15 M$\Omega$ cm, model Essential, Millipore, UK) and placed on spawn bed, see Fig. 1b. The plastic container was placed inside a polypropylene bag (type 14A and 49.5 $\times$ 32 cm in size https://www.annforfungi.co.uk/shop/100-spawn-bags/) fitted with $0.5\,\upmu \hbox {m}$ air filter patch (Ann Miller’s Speciality Mushrooms, UK) and sealed with food storage clip (model Bevara, IKEA, UK). The insole was kept at ambient room temperature 18–22 $^{\circ }\hbox {C}$ inside the growth tent (in darkness). The insole was checked for growth every 3 days and additional moisture was added as required (via manual spray bottle with de-ionised water) from a distance of c. 10 cm to maintain required humidity levels for keeping the insole healthy. The insole was kept within the sterile tent at all times and any sources of contamination were kept away from the tent. After c. 3 weeks, uniform mycelium growth throughout the capillary matting was observed, see Fig. 1b. The colonised insole was carefully lifted off the spawn bed, see Fig. 1c.

**Figure 2:**
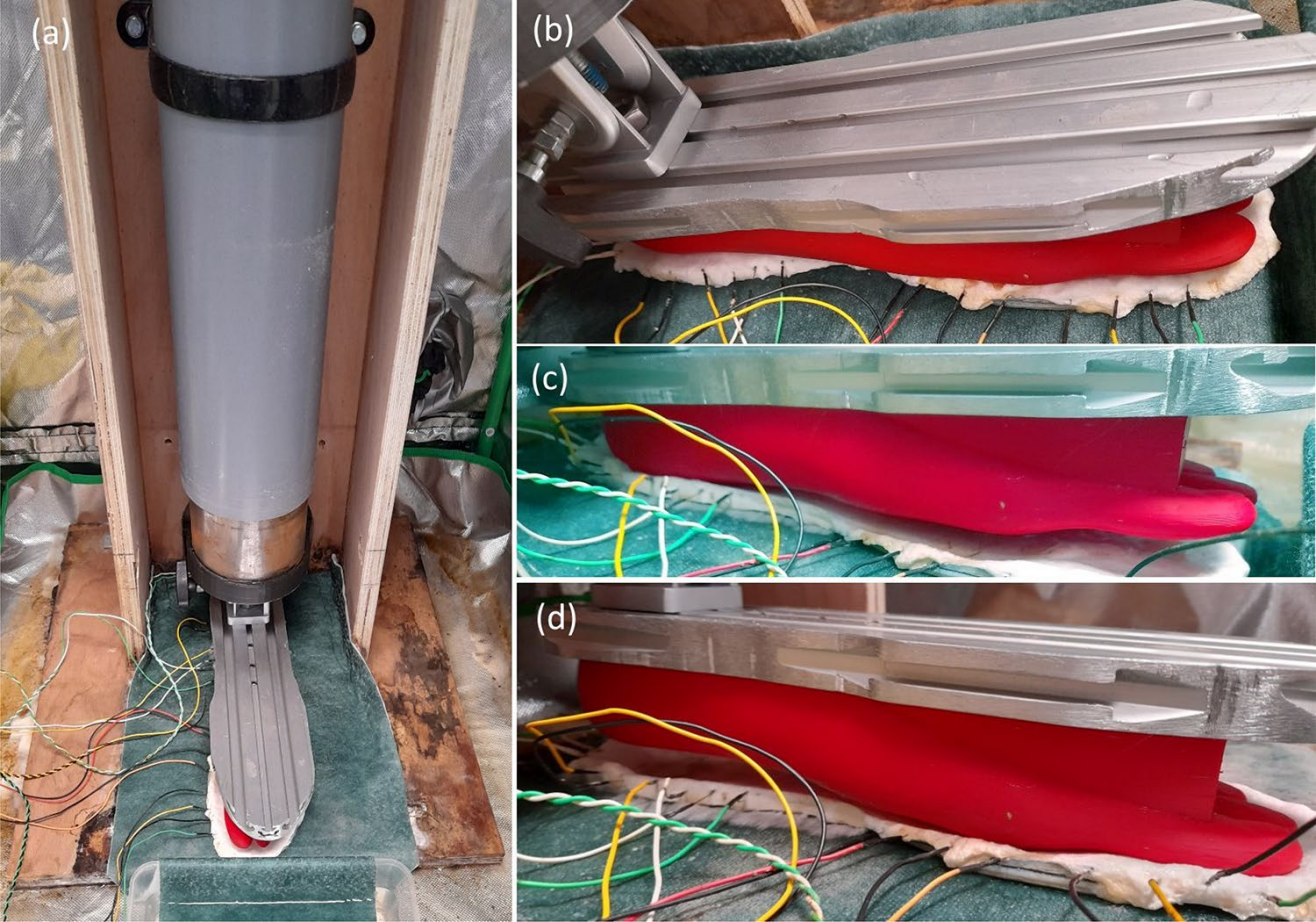
Bespoke insole test rig (**a**) setup inside growth tent (**b**) weight uniformly distributed via pivot joint on prosthetic foot (**c**) heel bias (**d**) toes bias.

A bespoke test rig was developed to apply compressive loading to insoles to replicate the weight of a human when walking and standing. A prosthetic foot (ladies, UK size 10) was 3D printed in acrylonitrile butadiene styrene (with Ultimaker S5, UK), see Fig. 2. The top part of the prosthetic foot was intentionally printed flat. A pivot joint (with integrated locking mechanism) was positioned between the weight and aluminium plate on top of the prosthetic foot to provide control over how the compressive load was distributed across the insole, see Fig. 2b. Test rig frame was assembled from plywood sheet (18 mm thickness) with plastic pipe (110 mm diameter). Mild steel bar (100 mm diameter) was free to move vertically inside the pipe to provide compressive loading on the insole at the bottom. For example, 500 mm length of steel bar weighs 35 kg which approximates a woman (with size 10 feet) standing on two feet (two 500 mm lengths can be used to replicate standing on one foot). The weight(s) could be raised and locked in the retracted position (by manual winch, fitted with a ratchet locking mechanism) to enable insoles to be interchanged and the load varied. To prevent the colonised insole slowly dehydrating over time (which might affect measurements) a sheet of capillary matting was placed under the insole, see Fig. 2a. The end of the capillary matting was left in a tray of de-ionised water which provided a source of moisture.The test rig was sterilised with 80% ethanol and placed in a sterilised hydroponic growing tent with a silver Mylar light proof inner lining (Green Box Tents, UK). Before and after the performance of each experiment, the rig and tent were sterilised with 80% ethanol.

Three modes of compressive loading were explored (i) toe bias, Fig. 2d (ii) heel bias, Fig. 2c and (iii) uniformly distributed, Fig. 2b.

Electrical activity of the mycelium colonising insoles was recorded using eight pairs of stainless steel sub-dermal needle electrodes (Spes Medica S.r.l., Italy), with twisted cables and ADC-24 (PICO Technology, UK) high-resolution data logger with a 24-bit A/D converter, galvanic isolation and software-selectable sample rates. The pairs of electrodes were pierced through the insole’s edge as shown in Fig. 2. We recorded electrical activity one sample per second. During the recording, the logger has been doing as many measurements as possible (typically up to 10 per second) and saving the average value. We set the acquisition voltage range to 156 mV. Each pair of electrodes, called a channel (Ch), reported a difference of the electrical potential between the electrodes. Distance between electrodes was 1–2 cm.

Numerical modelling of the electrical activity was implemented as follows.

**Figure 3:**
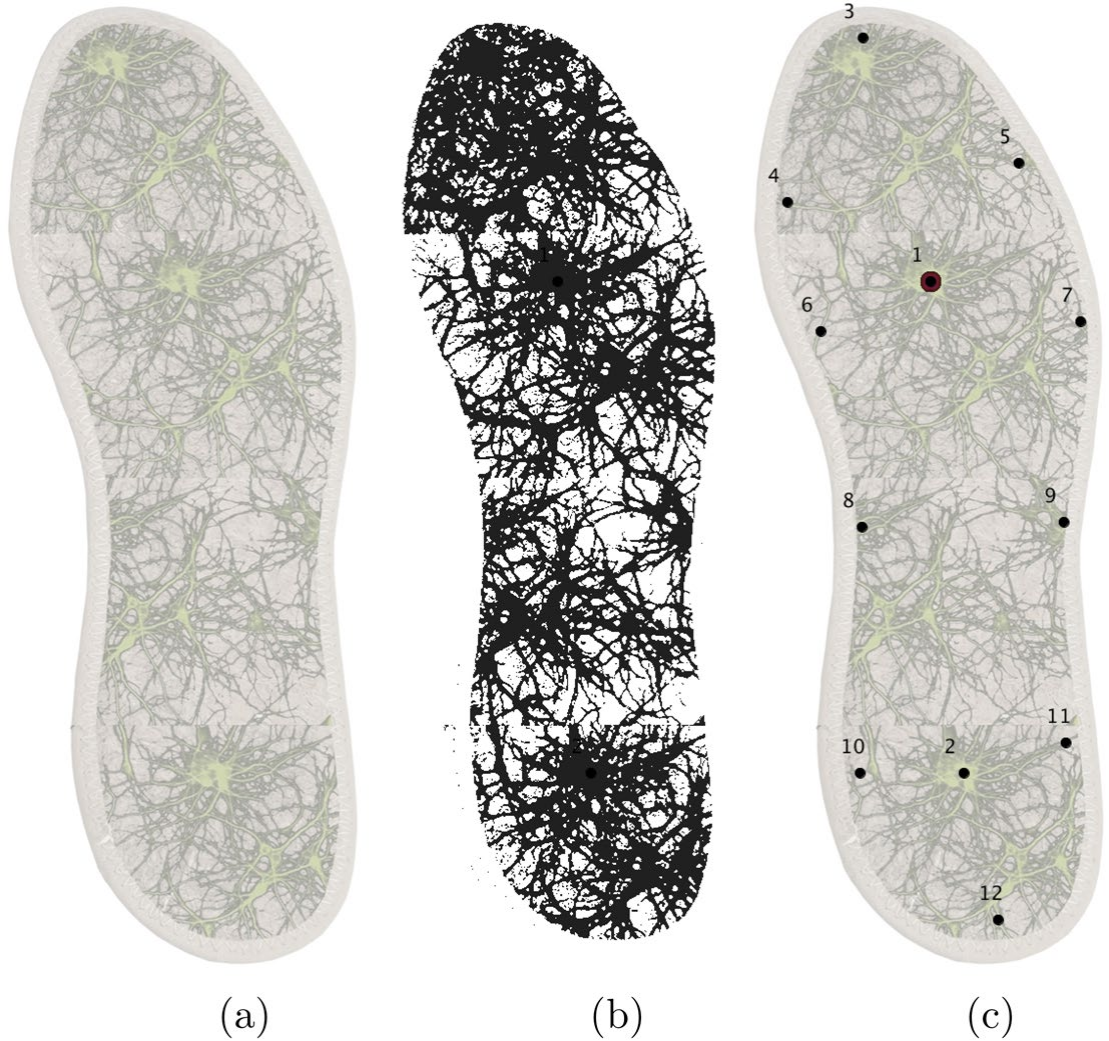
Image of the fungal colony, $1000 \times 960$ pixels used as a template conductive for modelling propagation of excitation in FitzHugh–Nagumo (FHN). (**a**) Original image, mycelium is seen as green pixels. (**b**) Conductive matrix *C*, conductive pixels are black. (**c**) Configuration of electrodes.

We used an artistic image of the mycelium network (Fig. 3a) projected onto a $364 \times 985$ nodes grid. The image was an integrative re-drawing of mycelium networks already grown in a surface. The original image $M=(m_{ij})_{1 \le j \le n_i, 1 \le j \le n_j}$, $m_{ij} \in \{ r_{ij}, g_{ij}, b_{ij} \}$, where $n_i=364$ and $n_j=985$, and $1 \le r, g, b \le 255$ (Fig. 3a), was converted to a conductive matrix $C=(m_{ij})_{1 \le i,j \le n}$ (Fig. 3b) derived from the image as follows: $m_{ij}=1$ if $r_{ij}>170$, $g_{ij}>170$ and $b_{ij}<200$; a dilution operation was applied to *C*.

FitzHugh–Nagumo (FHN) equations^17–19^ is a qualitative approximation of the Hodgkin–Huxley model^20^ of electrical activity of living cells:

1 $$\begin{aligned} \frac{\partial v}{\partial t}= & {} c_1 u (u-a) (1-u) - c_2 u v + I + D_u \nabla ^2 \end{aligned}$$

2 $$\begin{aligned} \frac{\partial v}{\partial t}= & {} b (u - v), \end{aligned}$$

where *u* is a value of a trans-membrane potential, *v* a variable accountable for a total slow ionic current, or a recovery variable responsible for a slow negative feedback, *I* is a value of an external stimulation current. The current through intra-cellular spaces is approximated by $D_u \nabla ^2$, where $D_u$ is a conductance. Detailed explanations of the ‘mechanics’ of the model are provided in^21^, here we shortly repeat some insights. The term $D_u \nabla ^2 u$ governs a passive spread of the current. The terms $c_2 u (u-a) (1-u)$ and $b (u - v)$ describe the ionic currents. The term $u (u-a) (1-u)$ has two stable fixed points $u=0$ and $u=1$ and one unstable point $u=a$, where *a* is a threshold of an excitation.

We integrated the system using the Euler method with the five-node Laplace operator, a time step $\Delta t=0.015$ and a grid point spacing $\Delta x = 2$, while other parameters were $D_u=1$, $a=0.13$, $b=0.013$, $c_1=0.26$. We controlled excitability of the medium by varying $c_2$ from 0.05 (fully excitable) to 0.015 (non excitable). Boundaries are considered to be impermeable: $\partial u/\partial \textbf{n}=0$, where $\textbf{n}$ is a vector normal to the boundary.

To record dynamics of excitation in the network, as if in laboratory experiments, we simulated electrodes by calculating a potential $p^t_x$ at an electrode location *x* as $p_x = \sum _{y: |x-y|<2} (u_x - v_x)$. Configuration of electrodes $1, \ldots , 16$ is shown in Fig. 3c.

To imitate a pressure onto insole we perturbed the medium around electrodes $E_1$ or $E_2$ or both.

Time-lapse snapshots provided in the paper were recorded at every 100th time step, and we display sites with $u>0.04$; videos and figures were produced by saving a frame of the simulation every 100th step of the numerical integration and assembling the saved frames into the video with a play rate of 30 fps. Videos are available at https://doi.org/10.5281/zenodo.5091807.

To examine the difference in responses of the insoles in more detail, the distribution of the weight across the insole was varied and applied in different regions (i.e., toe, heel, whole). That was designed in order to be similar to the way people change their weight distribution from heel to toe while walking. The number of spikes in each time period were automatically counted by utilising SciPy, an open-source collection containing mathematical algorithms and functions built on an extension of Python (https://docs.scipy.org). In specific, the function *find_peaks* was utilised to identify peaks with a prominence of 0.03 *mV* for this application. Then, a custom program was developed in Python to calculate the time difference between two spikes. Also, data visualization software Tableau was used to identify the potential (*mV*) differences under no load, even load, heel biased and toe biased load for every channel separately.

## Results

**Figure 4:**
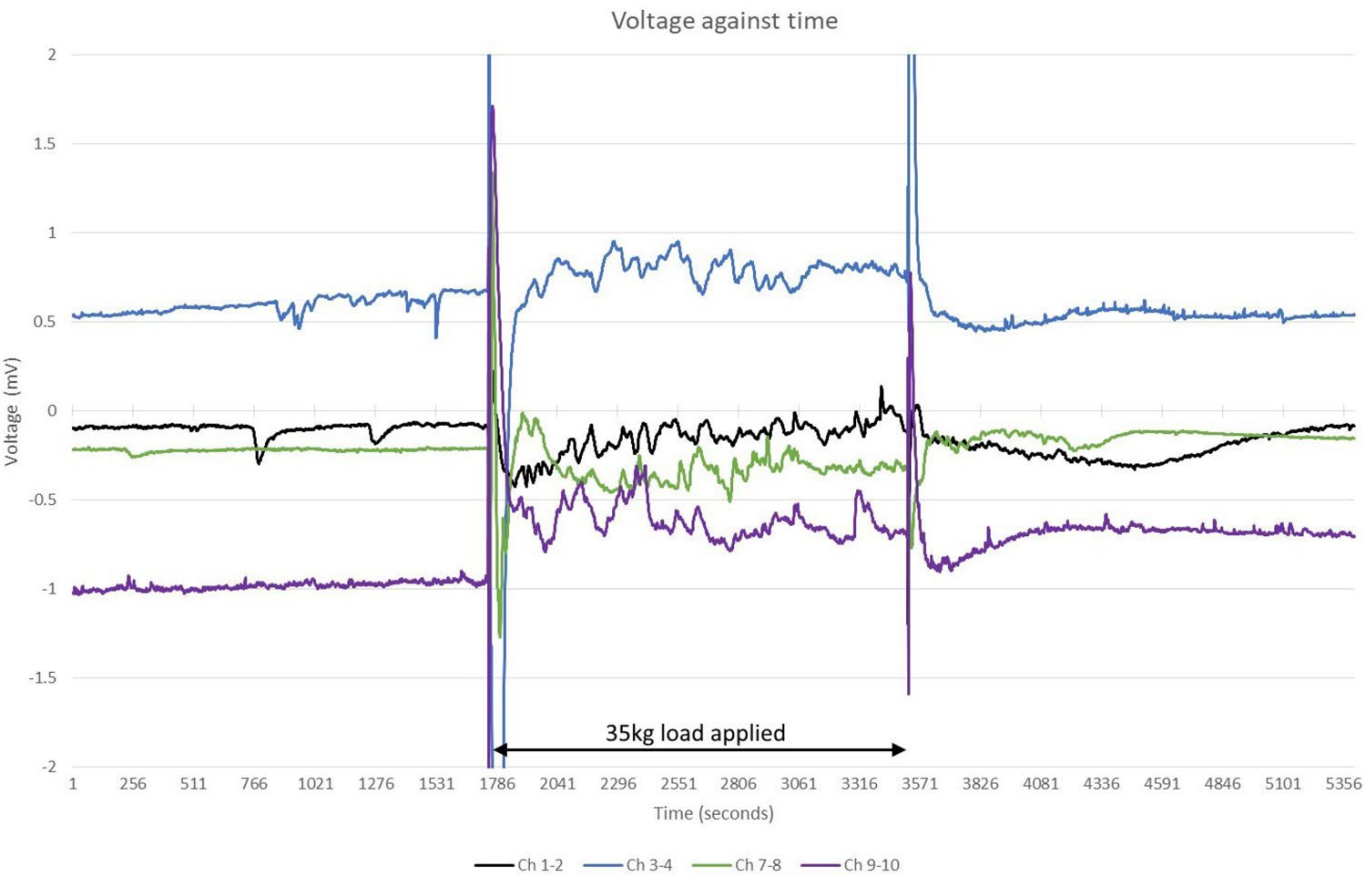
Electrical activity (recorded on Ch 1–2, Ch 3–4, Ch 7–8, Ch 9–10) before, during and after load (35 kg) was evenly distributed across insole.

Electrical activity was recorded for $\sim$ 30 min immediately before, $\sim$ 30 min during and $\sim$ 30 min immediately after 35 kg was evenly distributed across the insole, see Fig. 4.

**Figure 5:**
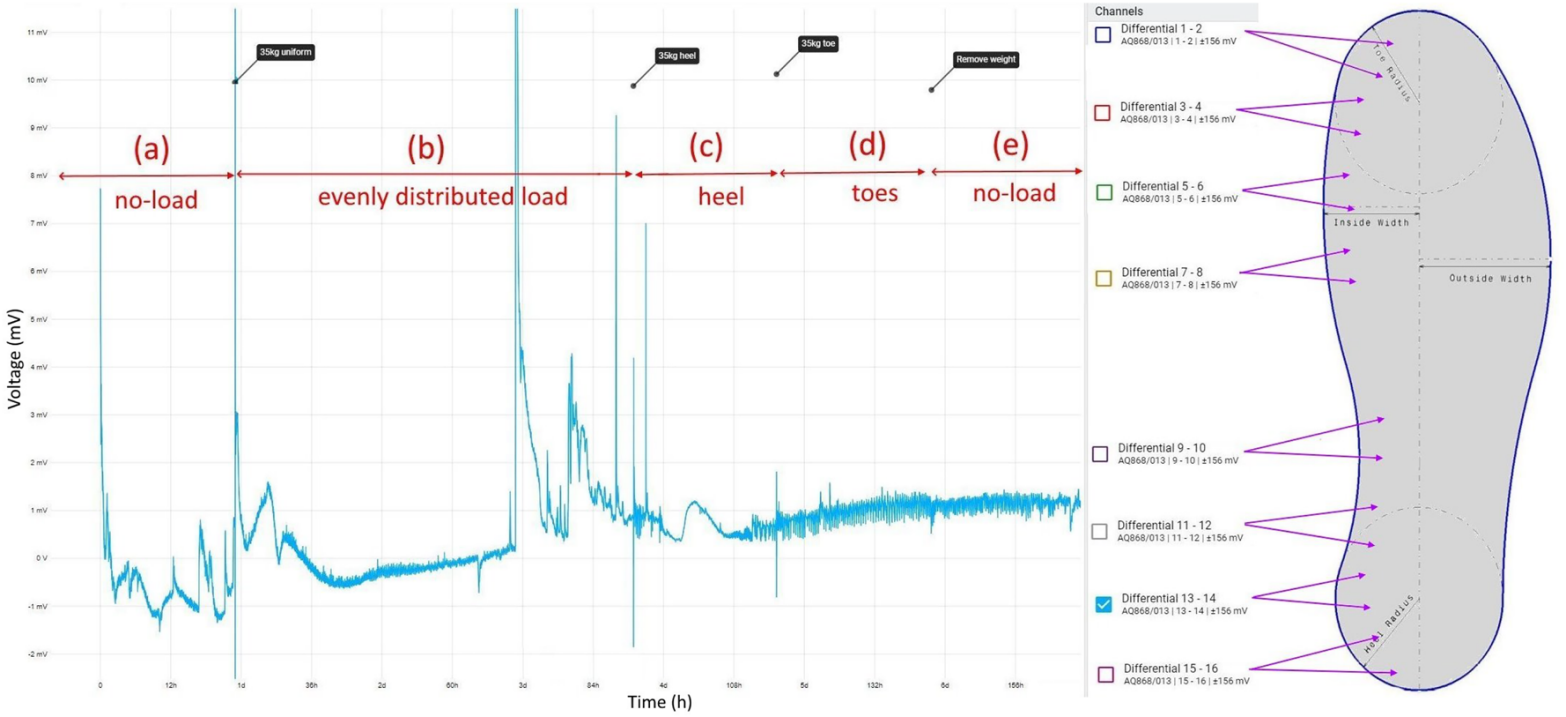
Exemplar of electrical activity recorded on Ch 13–14 (**a**) no load (**b**) load (35 kg) evenly distributed (**c**) load biased on heel region (**d**) load biased on toe region (**e**) no load.

Electrical activity was recorded for $\sim$24 h immediately before 35 kg was evenly distributed across the insole for $\sim$72 h. The prosthetic foot was then tilted back to bias the weight onto the heel region of the insole for $\sim$24 h. The prosthetic foot was then tilted forward to bias the weight onto the toe region of the insole for $\sim$24 h. Finally, the weight was removed for $\sim$24 h. Eight pairs of needle electrodes were distributed along the side of the insole as shown in Fig. 5.

**Figure 6:**
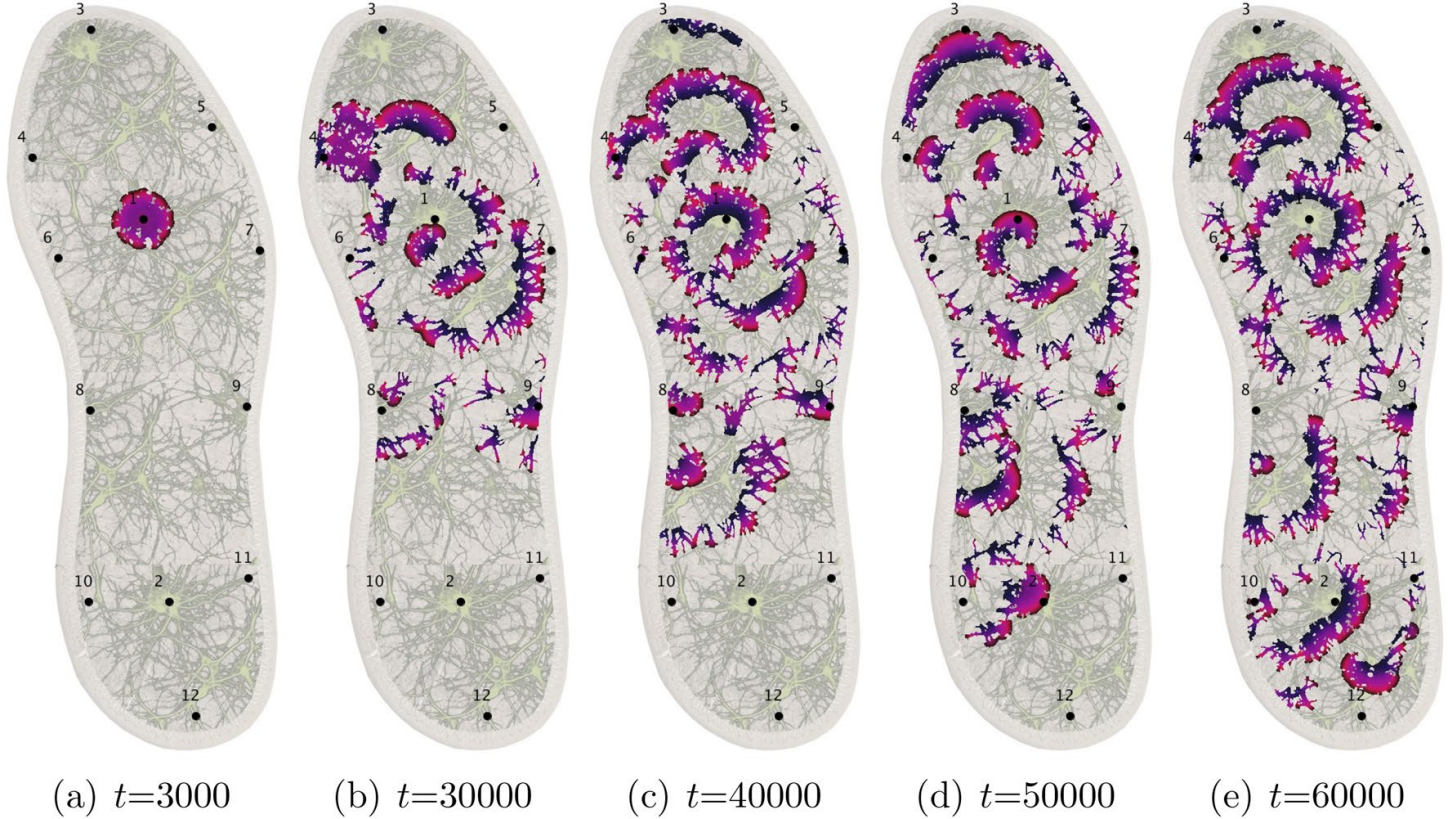
Snapshots of the excitation dynamics of the mycelium network colonising the insole produced from the computer simulations of FHN. Area around electrode $E_1$ has been excited originally.

**Figure 7:**
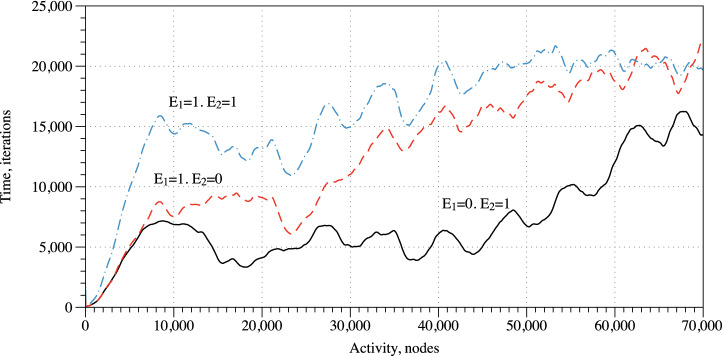
Activity of the mycelium network, for initial scenarios of excitation around electrode $E_2$, solid black, $E_1$, dashed red, $E_1$ and $E_2$, dot-dashed blue. The activity is measured in a number of nodes *x* with $u_x>0.1$.

When a small area, c. 10 nodes, is perturbed the excitation starts propagating along the simulated mycelium network (Fig. 6a,b). With time, typically after 50–60 K iterations of the integration, the excitation wave fronts span all the mycelium network (Fig. 6c,d,e). Due to inhomogeneity of the network source so the spiral waves are formed. They become sources of oscillatory excitations. Repeated propagation of the spiral waves is reflected in the oscillatory activity levels on the mycelium network (Fig. 7).

Further we will demonstrate that it is possible to discern what loci of the insole pressure was applied based on electrical activity of the mycelium network. As evidenced in Fig. 7 the overall activity of the mycelium network depends on the site of pressure application (as imitated via exciting areas around electrodes $E_1$ or $E_2$ or both). When consider the three possible scenarios of the stimulation, the overall activity is lowest when area around electrode $E_2$ is excited ($E_1=0, E_2=1$). This is due to relatively lower number of mycelium strands in that part of the insole. The overall activity increases in the scenario where area round $E_1$ is excited ($E_1=1, E_2=0$). And the highest level of overall activity is evidenced for the scenario when areas around both electrodes ($E_1=1, E_2=1$).

**Figure 8:**
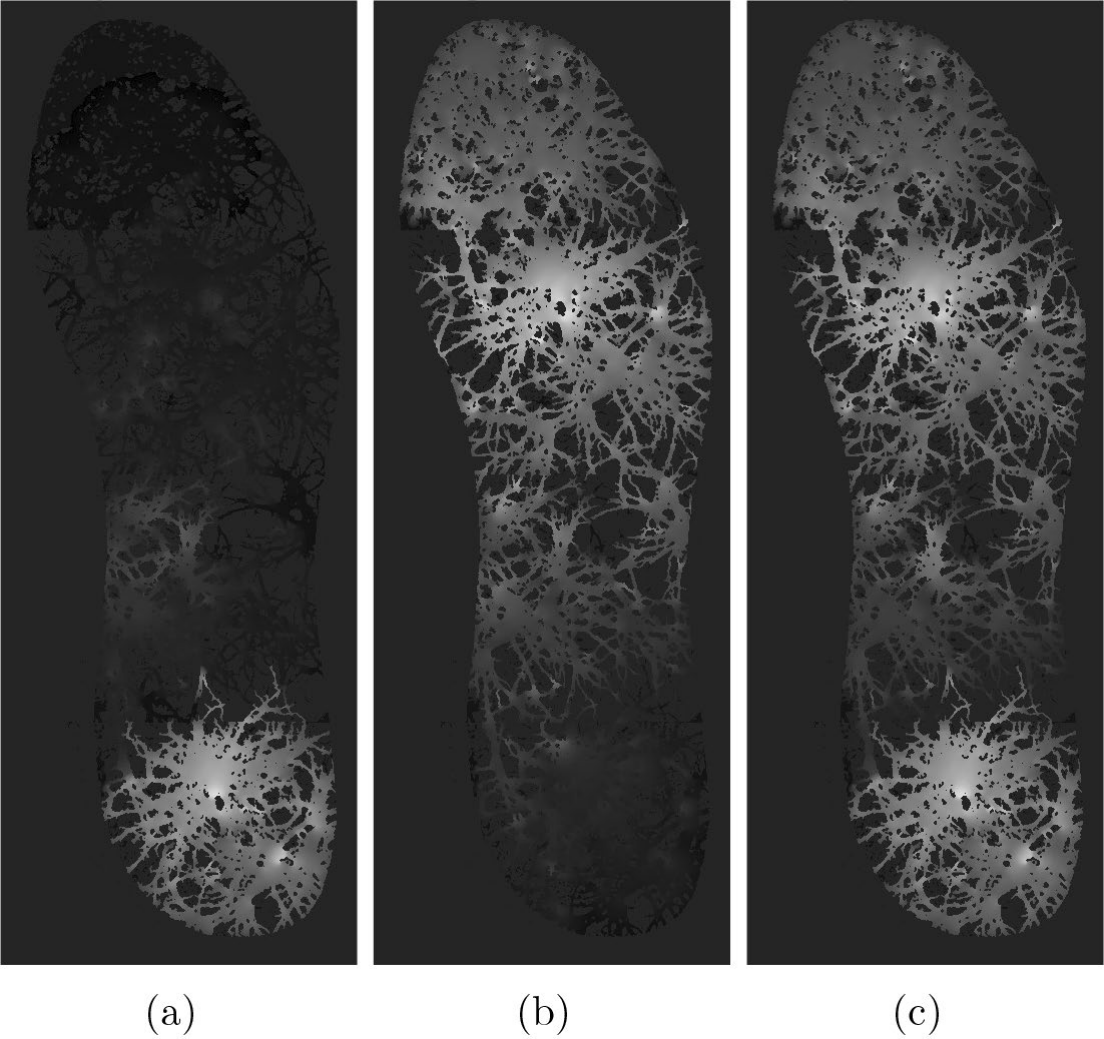
Coverage frequency, expressed in gradation of black. Areas never covered by excitation wave-fronts are black, areas covered most frequently are white. (**a**) Area around electrode $E_2$ have been excited initially. (**b**) Area around electrode $E_1$ have been excited initially. (**c**) Areas around electrodes $E_1$ and $E_2$ have been excited initially.

Coverage frequency could be another indicator to discern geometry of pressure. A coverage frequency at node *x* is a number of iterations values of excitation variable $u_x$ exceeded 0.1, normalised by maximum coverage frequency amongst the nodes. The coverage frequency is illustrated in Fig. 8. The coverage frequency is maximum around the areas of pressure application and might even reflect a distance, not an Euclidean distance but a distance in the propagation metric of the mycelium networks, form the pressure application site.

**Figure 9:**
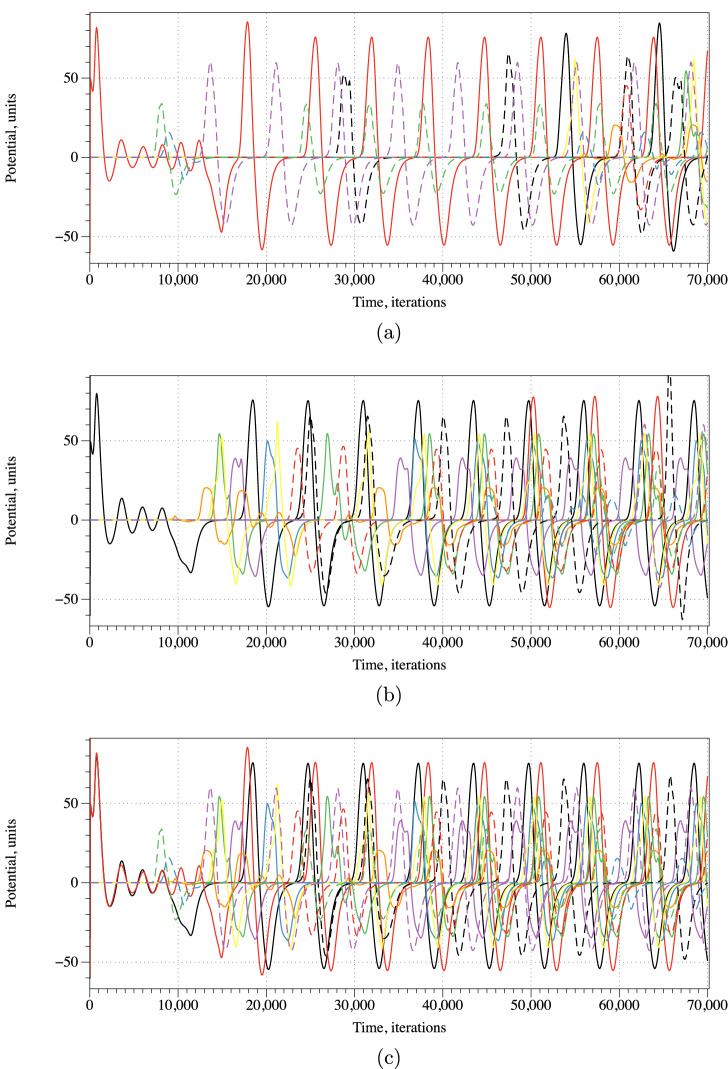
Potential recorded on electrodes $E_1, \ldots , E_{12}$ for initial scenarios of excitation around electrode (**a**) $E_2$, (**b**) $E_1$, (**c**) $E_1$ and $E_2$.

A third measure applied to discern geometry of pressure would be spiking activity recorded on the electrodes. Examples of the spiking for all three scenarios of pressure application are shown in Fig. 9. The patterns of spiking activities might give us unique representations of the geometries of pressure applications. The formal representation of the spiking patterns could be done by distributions of Boolean gates in the spiking activity. This original technique has been developed by us in frameworks of cytoskeleton networks^22^, fungal colony^23^ and ensemble of proteinoid microspheres^24^.

**Table 1:**
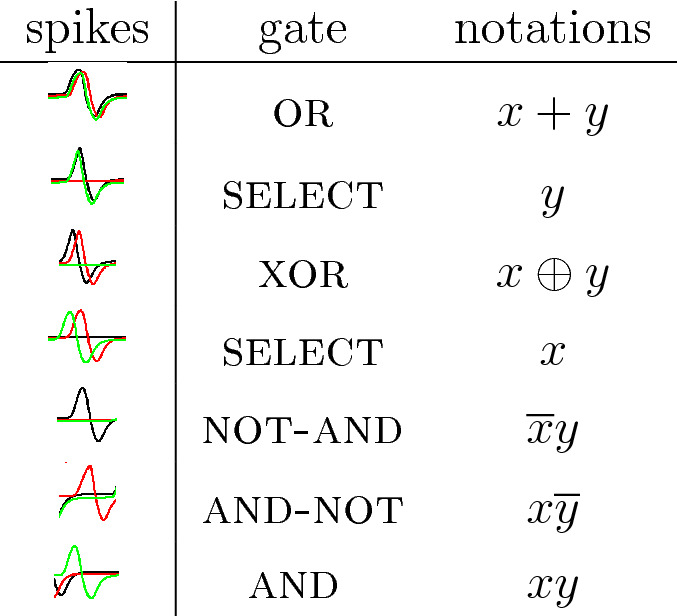
Representation of gates by combinations of spikes. Black lines show the potential when the network was stimulated by input pair (01), red by (10) and green by (11). Adamatzky proposed this representation originally in^22^.

A spiking activity of the mycelium network shown in Fig. 9 in a response to stimulation, i.e. application of inputs $(E_1,E_2)=\{ (0,1), (1,0), (1,1) \}$ via impulses at the electrodes $E_1$ and $E_2$, recorded from electrodes $E_1, \ldots , E_{12}$. We assume that each spike represents logical True and that spikes occurring within less than $2 \cdot 10^2$ iterations are simultaneous. Then a representation of gates by spikes and their combinations can be implemented as shown in Table 1. By selecting specific intervals of recordings we can realise several gates in a single site of recording. In this particular case we assumed that spikes are separated if their occurrences are more than $10^3$ iterations apart. In the simulated scenarios, we found that the following Boolean functions can be implemented on the electrodes $E_1, \ldots , E_{12}$. Three or gates are realised on electrodes $E_3$, $E_8$ and $E_{12}$. Ten Select*y*, where *y*=true signifies initial excitation around electrode $E_2$, are realised on electrodes $E_3$ and $E_{12}$. Fifty Select*x*, where *x*=true signifies initial excitation around electrode $E_1$, are realised on the electrodes but $E_1$, $E_9$ and $E_{11}$. Five not-and gate, in the form not *x* and *y*, are realised on electrodes $E_2$, $E_9$ and $E_{10}$. The implementation of logical functions on the electrodes will allow for logical inference about geometries of pressure applied to insoles.

## Discussion

Initially, off-the-shelf hemp insoles were used for inoculation with fungus *Pleurotus ostreatus*. The hemp insoles were sourced from different manufacturers (Hemp Made In UA, Ukraine, collection 100% natural hemp insoles) (HanfHaus, Denmark, collection organic hemp insoles) (Hemps, Ukraine, collection organic hemp multilayer therapeutic insoles with wormwood) (The Hemp Shop, Somerset, collection hemp organic insoles) and showed poor fungal colonisation, possibly due to unknown chemical processes to minimise bacterial growth which could lead to undesirable foot wear odour. Further testing of bespoke insoles cut from non-woven hemp matting (manufactured by Pemmiproducts, Germany) showed improved colonisation by fungi but inconsistent electrical activity (possibly due to inconsistent moisture level) and weak mechanical robustness. Capillary matting was tested and identified compatible with the biological organism being utilised and a strong candidate for the desired functionality.

It was observed that the risk of unwanted bacterial infection during colonisation of the insole could be reduced by keeping the insole inside a sealed local environment (for example, plastic bag fitted with sub-micron air filter) that mitigates airborne infection. Additionally, enclosing in a bag helps to prevent the insole dehydrating by maintaining a high local air humidity. Keeping the insole in darkness or low intensity light encourages its colonisation.

Optionally, forming the insole from two sheets of capillary matting allows a sandwich to be formed with nutrient layer (such as Rye grain seeds) between the top and bottom layer. This can allow the fungi to remain active for longer. Insoles infused with flour paste were prone to infection (even with sterilisation via autoclave).

The large internal volume and porous seals (long fabric zips) on the growth tent containing test rig was prone to low humidity, even with an open container of water present. It was found adding a sheet of capillary matting under the insole, see Fig. 2a, helped to maintain an adequate moisture level in the insole for fungal activity. The end of the sheet was left in a tray of de-ionised water which provided a source of moisture.

Oscillations in plant membrane are already known^25^. The physiological role of such oscillations has been the subject of much speculation. It has been hypothesised these oscillations are links to plants’ adaptive response to environmental stresses^26^.

The number of spikes (< 0.1 mV) recorded over three $\sim$30 m periods before, during after even compressive load are summarised in Table 2. Further, it was observed that periodicity of electrical spikes changed when the mycelium was under compressive load.

**Table 2:** Electrical response to weight (electrical spikes $<0.1\,\hbox {mV}$).

Differential channels	30 min period before weight	30 min period during weight	30 min period after weight
Ch 1–2	2	10	1
Ch 3–4	2	6	0
Ch 7–8	0	8	3
Ch 9–10	0	7	2

Measurements indicate a layer of mycelium integrated into an insole shows electrical response to mechanical stimulation with change in oscillatory activity. In particular, the number of spikes increases under compressive load. The response to removing weight is different to applying weight.

**Figure 10:**
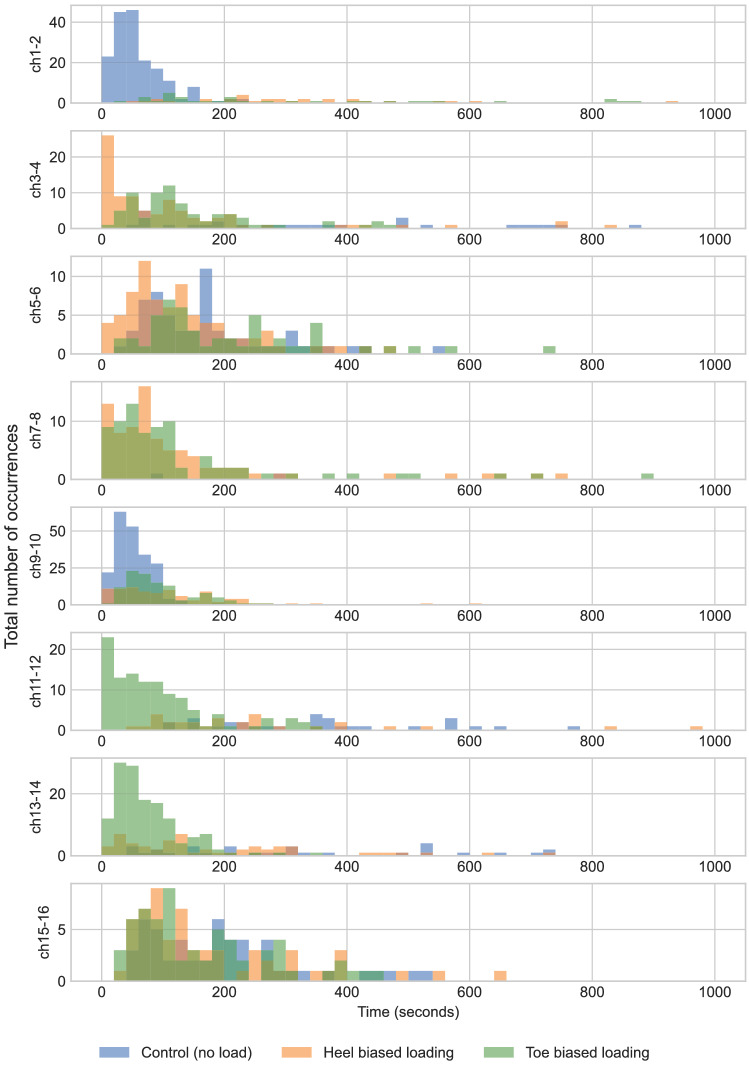
Histograms of the distribution of time differences between spikes in each insole region under a range of load condition.

**Figure 11:**
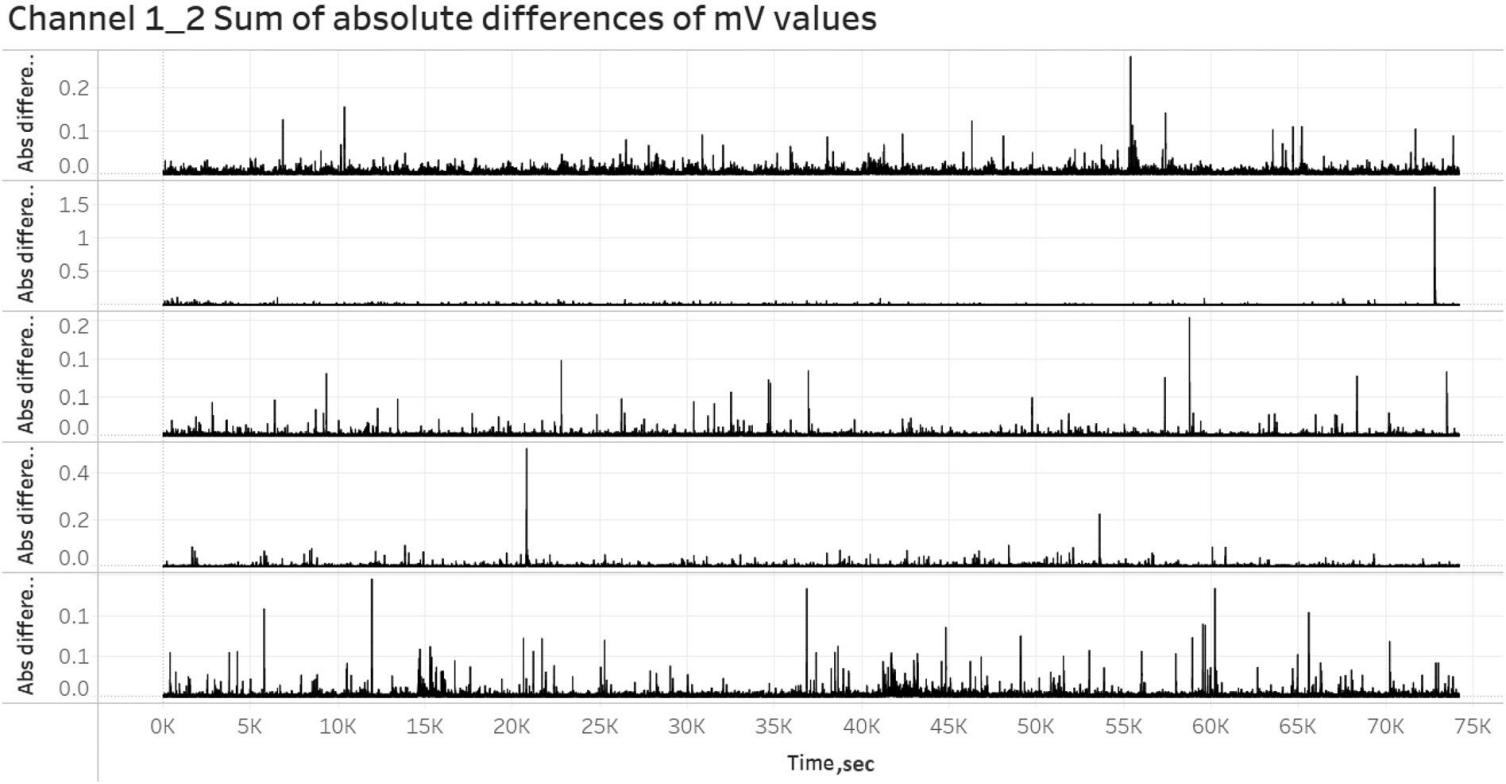
Graph showing decrease of amplitude when under even load compared to no load application for Channels 1–2.

**Figure 12:**
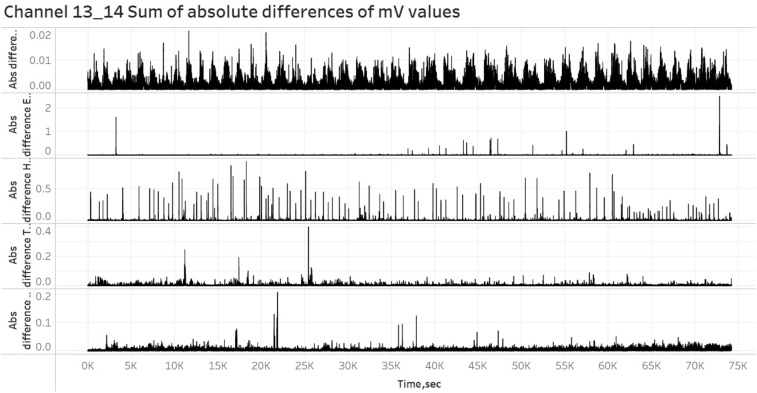
Graph showing decrease of amplitude when under even load compared to no load application for Channels 13–14.

To examine the response of the distribution of the weight across the insole (i.e., toe, heel, whole, similar to the way people change their weight distribution from heel to toe while walking) the data were compared. Histograms of the distribution of time differences between spikes in each insole region under no load, heel biased and toe biased load were produced and they were drawn over the same axes to allow the comparison of the results as shown in Fig. 10. Moreover, the visualization to identify the potential differences under no load, even load, heel biased and toe biased load for every channel separately is shown in Figs. 11 and 12. The amplitude of spikes decreases under even load application compared to no load application. No conclusions could be made regarding the electrical activity under heel biased and toe biased load. It was therefore, not possible to accurately discern the distribution of compressive loading across the insole from analysis of electrical responses.

Experimentation identified the rate of occurrence of spikes is lower than desirable to accurately infer the weight bearing when walking. However, when standing for a period, electrical activity can be collected and analysed to infer weight bearing which can be used for anatomical diagnostics^27,28^. Continuous monitoring of feet could offer numerous medical/health benefits^29–31^. For example, early detection of health related conditions (such as knee injury) or tiredness. It can also be useful for sports training^32–34^.

In the experimental setup described, sensory mapping is limited to ‘1.5D’ (8 pairs of differential electrodes in a row and limited vertical motion) however this could be expanded to ‘2.5D’ by mapping with a 2D distributed array of electrodes across the insole. For example, needle electrodes replaced by thin/flexible wires integrated into the capillary matting such that uninsulated sections of the wires are spatially distributed and electrical connections are realised to the edge of the insole.

Direct conversion of mechanical energy into electricity offers potential as power source for various systems^35–37^.

It was observed during the fabrication and testing of a diverse range of prototype wearables (including clothing) that capillary matting offer superior durability over hemp matting (in particular on flexible clothing joints, knees and elbows) and easy of interfacing to conventional fabrics (sewing and gluing). Therefore, capillary matting might be useful substrate for a range of smart fungal wearables.

Smart footwear offers benefits to safety footwear^38^. For example, automatic notification of injury to user and emergency services. Awareness of foot activity might also be beneficial in various environments such as driving (for example, enabling the vehicle to respond before the driver’s foot has touched a pedal).

Simulation using FitzHugh–Nagumo model numerically illustrated how excitation wave-fronts behave in a mycelium network colonising an insole and shown that it is possible to discern pressure points from the mycelium electrical activity.

## Conclusions

Electrical activity (spiking) was recorded in mycelium bound composites fabricated into insoles. The number and periodicity of electrical spikes change when the mycelium is subjected to compressive loading. We have shown that it might be possible to discern the loading from the electrical response of the fungi to stimuli. The results advance the development of intelligent sensing insoles which are a building block towards more generic reactive fungal wearables. Electrical activity changes in both spatial and temporal domains. Using FitzHugh–Nagumo model we numerically illustrated how excitation wave-fronts behave in a mycelium network colonising an insole and shown that it might be possible to discern pressure points from the mycelium electrical activity. Fungal based insoles offer augmented functionality (sensory) and aesthetic (personal fashion). We presented results of scoping experiments on living biowearables. The results open new horizons in exploring feasibility of living fungal materials in everyday life. Directions of future research will involve bench marking of the prototypes and testing them in real life.

## Data Availability

The datasets made and analysed during the current study are available from the corresponding author on reasonable request.
